# Molecular Precursor Engineering of Lignin-Derived Carbon Dots for Multicolor Fluorescence and Metal-Ion Sensing

**DOI:** 10.3390/nano16150931

**Published:** 2026-07-28

**Authors:** Bole Ma, Huiqing Wei, Jiaqi Tan, Liheng Chen, Minting Liang, Xueqing Qiu

**Affiliations:** 1 Guangdong Provincial Key Laboratory of Plant Resources Biorefinery, School of Chemical Engineering and Light Industry, Guangdong University of Technology, Guangzhou 510006, China; mabole0225@163.com (B.M.);; 2 Guangdong Provincial Laboratory of Chemistry and Fine Chemical Engineering Jieyang Center, Jieyang 515200, China; 3 Guangdong Basic Research Center of Excellence for Ecological Security and Green Development in Guangdong-Hong Kong-Marco Greater Bay Area (GBA), Guangdong University of Technology, Guangzhou 510006, China

**Keywords:** lignin, carbon dots, solvent-free synthesis, precursor engineering, photoluminescence, metal-ion sensing

## Abstract

Lignin-derived carbon dots (CDs) are promising sustainable fluorescent nanomaterials for environmental sensing, yet precise regulation of their emission behavior and ion-recognition selectivity remains challenging. Herein, a solvent-free precursor–structure–engineering strategy was developed to prepare lignin-derived CDs with tunable photoluminescence and selective metal-ion sensing. Industrial alkali lignin, lysine, and oxalic acid were used as the carbon source, nitrogen source, and carbonization promoter, respectively, while cysteine, histidine, and p-phenylenediamine were introduced as functional precursors. The resulting C-CDs, H-CDs, and P-CDs showed distinct optical and sensing properties. H-CDs exhibited the highest photoluminescence quantum yield of 50.98%, attributed to enhanced graphitic nitrogen formation and electronic conjugation. P-CDs displayed a red-shifted emission at approximately 573 nm due to extended π-conjugated domains. Moreover, C-CDs, H-CDs, and P-CDs showed preferential fluorescence responses toward Fe^3+^, Cu^2+^, and Ag^+^, with detection limits of 0.26, 0.05, and 0.11 μM, respectively. The quenching behavior was inconsistent with a dominant dynamic collisional process and was instead associated primarily with metal–surface interactions. This work clarifies the precursor–structure–property relationship and provides a sustainable route for designing lignin-derived fluorescent probes.

## 1. Introduction

Carbon dots (CDs) are a class of zero-dimensional fluorescent carbon nanomaterials with typical sizes below 10 nm [1,2]. Owing to their tunable photoluminescence, good water dispersibility, low cytotoxicity, chemical stability, and abundant surface functional groups, CDs have attracted broad interest in fluorescence sensing, bioimaging, anti-counterfeiting, photocatalysis, and environmental monitoring [3,4]. In particular, CD-based fluorescent probes are highly promising for metal-ion detection because their surface oxygen-, nitrogen-, and sulfur-containing groups can interact with metal ions and induce measurable photoluminescence changes [5,6,7]. However, practical sensing applications still require CDs with controllable emission wavelengths, high fluorescence efficiency, good environmental stability, and specific ion-recognition capability.

Considerable efforts have been made to regulate the optical and sensing properties of CDs through heteroatom doping, surface passivation, molecular precursor design, and reaction-condition control [8,9]. Nitrogen- and sulfur-containing precursors have been widely employed to introduce surface-active sites and modulate the electronic structure of CDs, thereby improving their fluorescence response toward metal ions [10,11]. Pan et al. prepared nitrogen- and sulfur-codoped CDs using lignin as the carbon source and 2-thiazoline-2-thiol as the nitrogen and sulfur source, demonstrating that heteroatom-containing precursors can enhance metal-coordination-related functionality in lignin-based carbon systems [12]. Phenylenediamine-derived CDs have also been used to regulate emission from short to long wavelengths, suggesting that extended conjugation, graphitic nitrogen formation, and carbon-core growth can reduce the energy gap and promote long-wavelength emission [13,14]. These studies indicate that precursor molecular structure plays a decisive role in directing the carbonization pathway, electronic structure, and photoluminescence behavior of CDs [15,16]. However, most precursor–engineering strategies have been developed using small-molecule carbon sources or relatively simple reaction systems. Extending these design principles to biomass-derived CDs, especially lignin-derived CDs, remains challenging because of the structural complexity and heterogeneous carbonization behavior of biomass precursors [17,18].

Lignin, as the most abundant renewable aromatic biopolymer, provides a particularly attractive platform for examining precursor–structure regulation in biomass-derived CDs [19,20,21]. Its rich aromatic units and oxygen-containing functional groups are favorable for constructing conjugated carbon domains and surface-active sites, while the conversion of industrial lignin into fluorescent CDs also offers a promising route for biomass valorization [22,23,24]. Previous studies on lignin-derived CDs have mainly focused on improving fluorescence intensity, introducing heteroatom dopants, or developing fluorescent probes for specific ions such as Fe^3+^ and Cu^2+^ [25,26]. These works have demonstrated the feasibility of using lignin as a carbon precursor for functional fluorescent nanomaterials [27,28]. Nevertheless, the intrinsic structural heterogeneity of lignin often leads to complicated carbonization pathways, making it difficult to precisely regulate emission wavelength, surface-state distribution, and sensing selectivity [29,30]. In particular, systematic studies on how different functional precursor structures modulate both photoluminescence behavior and metal-ion recognition in lignin-derived CDs are still limited. Recent reviews of green and tailored carbon dots have highlighted the importance of precursor selection, surface coordination groups, heterogeneous emissive states, aggregation behavior, and matrix interference in determining heavy-metal-ion sensing performance [31,32].

Herein, we report a solvent-free precursor–structure–engineering strategy for preparing lignin-derived CDs with tunable photoluminescence and selective metal-ion sensing capability. Industrial alkali lignin was used as the carbon source, lysine as the nitrogen source, and oxalic acid as the carbonization promoter. Cysteine, histidine, and p-phenylenediamine were selected as functional molecular precursors to modulate heteroatom configuration, conjugated domains, and surface coordination sites. The resulting C-CDs, H-CDs, and P-CDs exhibited distinct optical and sensing behaviors. H-CDs showed the highest photoluminescence quantum yield of 50.98%, mainly associated with enhanced graphitic nitrogen formation, whereas P-CDs displayed a red-shifted emission at approximately 573 nm due to the extended aromatic conjugation induced by p-phenylenediamine. Moreover, C-CDs, H-CDs, and P-CDs showed selective fluorescence responses toward Fe^3+^, Cu^2+^, and Ag^+^, respectively, with H-CDs achieving a low detection limit of 0.05 μM for Cu^2+^. This work establishes a structure–property relationship among precursor molecular structure, carbon-dot composition, fluorescence behavior, and ion-recognition performance, providing a sustainable approach for designing lignin-derived fluorescent nanomaterials for environmental sensing.

## 2. Experimental Section

### 2.1. Materials

Industrial alkali lignin was used as the carbon source and was obtained from Shandong Longlive Bio-technology Co., Ltd. (Yucheng, Shandong, China). l-Lysine, cysteine, histidine, and p-phenylenediamine, all of analytical grade, were purchased from Shanghai Macklin Biochemical Co., Ltd. (Shanghai, China). Oxalic acid, used as the foaming agent, was purchased from Sinopharm Chemical Reagent Co., Ltd. (Shanghai, China). Deionized or ultrapure water, as specified, was used throughout the experiments. The metal salts used in the ion-sensing experiments were prepared as aqueous stock solutions before use and included copper sulfate, copper chloride, silver nitrate, ferric chloride, ferrous chloride, anhydrous aluminum chloride, potassium chloride, sodium chloride, magnesium chloride hexahydrate, lead chloride, cobalt chloride hexahydrate, and nickel chloride. Unless otherwise specified, all metal salts were of analytical grade. Copper sulfate, copper chloride, silver nitrate, ferric chloride, anhydrous aluminum chloride, sodium chloride, lead chloride, cobalt chloride hexahydrate, and nickel chloride were purchased from Shanghai Aladdin Biochemical Technology Co., Ltd. (Shanghai, China). Ferrous chloride, potassium chloride, and magnesium chloride hexahydrate were purchased from Shanghai Macklin Biochemical Co., Ltd. (Shanghai, China). Hydrochloric acid was purchased from Guangzhou Chemical Reagent Factory (Guangzhou, China), and sodium hydroxide was purchased from Shanghai Macklin Biochemical Co., Ltd. (Shanghai, China).

### 2.2. Synthesis of Lignin-Derived Carbon Dots

Lignin-derived CDs were prepared through a solvent-free spatially confined carbonization method. Typically, lignin, lysine, oxalic acid, and a selected functional precursor were thoroughly ground in an agate mortar until a homogeneous solid mixture was obtained. The mixture was then transferred into a flask and heated in an oil bath at 180 °C for 6 h under atmospheric pressure.

For the preparation of C-CDs, lignin, lysine, oxalic acid, and cysteine were used at masses of 0.30, 1.50, 2.40, and 0.20 g, respectively. For H-CDs, lignin, lysine, oxalic acid, and histidine were used at masses of 0.60, 1.50, 2.40, and 0.60 g, respectively. For P-CDs, lignin, lysine, oxalic acid, and p-phenylenediamine were used at masses of 0.30, 1.50, 2.40, and 0.30 g, respectively. After the reaction, deionized water at 25 °C was added to disperse the carbonized products. The obtained dark dispersion was centrifuged at 10,000 rpm for 5 min to remove insoluble residues and large particles. The supernatant was dialyzed against deionized water using a dialysis membrane with a molecular weight cutoff of 1000 Da for 48 h. The purified dispersion was then filtered through a 100 nm microporous membrane and freeze-dried to obtain solid CD powders.

### 2.3. Characterization

The morphology and particle-size distribution of the CDs were characterized using a field-emission transmission electron microscope (Talos F200S, FEI Company, Hillsboro, OR, USA). Before TEM observation, the diluted CD aqueous dispersion was filtered through a 0.22 μm microporous membrane, dropped onto an ultrathin carbon-coated copper grid, and naturally dried. High-resolution TEM was used to observe the lattice structure of the CDs. UV–vis absorption spectra were recorded using a UV-8000S UV–vis spectrophotometer (Shanghai Metash Instruments Co., Ltd., Shanghai, China). For samples available as freeze-dried powders, the CD powders were dissolved in ultrapure water to prepare aqueous dispersions with a concentration of approximately 1 mg·mL^−1^. For samples without sufficient solid product, the dialyzed and filtered CD dispersions were directly diluted before measurement. For fluorescence quantum yield measurements, the absorbance of the sample solution at the excitation wavelength was controlled below 0.1. Photoluminescence spectra were recorded using a FluoroMax-4 spectrofluorometer (HORIBA Jobin Yvon, Edison, NJ, USA). Before measurement, the freeze-dried CD powders were dissolved in ultrapure water and treated by ultrasonication to obtain clear and homogeneous dispersions. Excitation spectra, emission spectra, and excitation–emission matrix spectra were collected to characterize the fluorescence behavior of the samples. Time-resolved fluorescence decay curves were recorded using the same excitation and emission wavelengths for each CD/metal-ion pair. The decay curves were fitted using a biexponential model, R(t) = B_1_exp(−t/τ_1_) + B_2_exp(−t/τ_2_), and the intensity-weighted average lifetime was calculated as τavg = ΣBiτi^2^/ΣBiτi. The fluorescence quantum yield was determined by a comparative method using quinine sulfate dissolved in 0.1 mol·L^−1^ H_2_SO_4_ as the standard reference. The quantum yield of quinine sulfate was taken as 55% under 350 nm excitation. The absorbance values of both the sample and reference solutions at the excitation wavelength were kept below 0.1. Fourier-transform infrared spectra were recorded using a Nicolet iS50R FTIR spectrometer (Thermo Fisher Scientific, Waltham, MA, USA). The dialyzed and filtered CD dispersions were freeze-dried before testing. The dried CD powders were mixed with spectroscopic-grade KBr, ground thoroughly, and pressed into transparent pellets for FTIR measurement. X-ray diffraction measurements were performed using a D8 ADVANCE X-ray diffractometer (Bruker AXS GmbH, Karlsruhe, Germany). X-ray photoelectron spectroscopy was performed using an ESCALAB 250Xi X-ray photoelectron spectrometer (Thermo Fisher Scientific, Waltham, MA, USA). For XPS measurement, the freeze-dried solid samples were directly used after pressing. High-resolution spectra of C 1s, N 1s, O 1s, and S 2p were recorded when applicable. Additional experimental details are provided in Sections S1–S6 of the Supplementary Material.

## 3. Results and Discussion

### 3.1. Precursor-Engineered Solvent-Free Synthesis and Photoluminescence Regulation

**Scheme 1:**
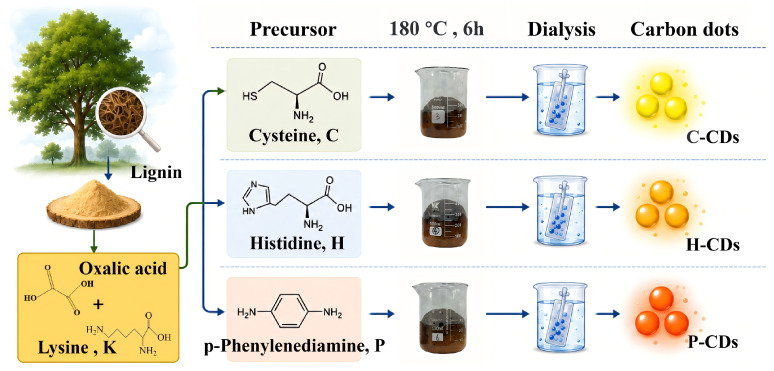
Flow chart of the solvent-free preparation of carbon dots.

A solvent-free spatially confined carbonization strategy was developed to prepare lignin-derived carbon dots (CDs) with tunable photoluminescence and selective metal-ion sensing capability. As illustrated in Scheme 1, the synthesis involved direct thermal treatment of a solid precursor mixture composed of industrial alkali lignin, lysine, oxalic acid, and a selected functional molecular precursor. Compared with conventional hydrothermal or solvothermal methods, this solvent-free process avoids the involvement of bulk solvent molecules during carbonization and provides a concentrated reaction environment [33]. Such a locally confined environment is beneficial for promoting dehydration, condensation, aromatization, and surface-state construction among the solid precursors [34].

In this system, industrial alkali lignin was used as the primary carbon source. Its abundant aromatic units provide a structural basis for the formation of sp^2^ carbon domains, while its oxygen-containing functional groups can participate in condensation and surface functionalization reactions [35]. Lysine was introduced as both a nitrogen source and a foaming-assisted component. During thermal treatment, lysine may undergo decomposition, dehydration, and condensation reactions, generating gaseous products and crosslinked intermediates [36]. These processes contribute to the formation of a foamed and spatially confined microenvironment, which helps restrict excessive aggregation of carbonized intermediates and favors the formation of nanoscale CDs. Oxalic acid acted as a carbonization promoter and acid-assisted condensation component, facilitating dehydration and carbonization of the precursor mixture.

On this basis, cysteine, histidine, and p-phenylenediamine were introduced to regulate the carbonization pathway, heteroatom configuration, conjugated structure, and surface coordination sites of the resulting CDs. The selection of these three molecules was based on their distinct molecular structures and electronic characteristics. Cysteine contains amino, carboxyl, and thiol groups, which can participate in condensation reactions and provide sulfur-containing surface sites for metal-ion coordination [37]. Histidine contains an imidazole heterocycle with relatively high thermal stability, which can serve as an effective nitrogen-containing structural unit during carbonization. The imidazole ring is expected to promote nitrogen incorporation into the carbon framework and favor the formation of graphitic nitrogen. In contrast, p-phenylenediamine possesses a rigid aromatic skeleton and two electron-donating amino groups, making it favorable for π-conjugation extension, charge-transfer interactions, and long-wavelength emission [38].

**Figure 1:**
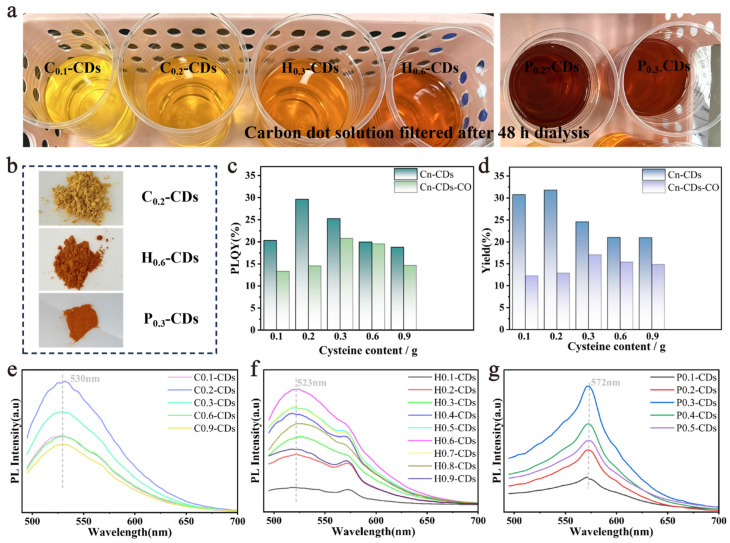
(**a**) Colors of the different carbon-dot dispersions after dialysis and filtration under natural light. (**b**) Photographs of the corresponding freeze-dried carbon-dot samples. Comparisons of (**c**) photoluminescence quantum yield and (**d**) product yield between C_x_-CDs prepared using the oil-bath-based spatially confined method and the corresponding C_x_-CDs-CO samples prepared using a constant-temperature oven. Photoluminescence emission spectra of (**e**) C_x_-CDs, (**f**) H_x_-CDs, and (**g**) P_x_-CDs with different functional-precursor loadings under 480 nm excitation. In the sample codes C_x_-CDs, H_x_-CDs, and P_x_-CDs, x denotes the mass, in grams, of cysteine, histidine, and p-phenylenediamine used in the precursor mixture, respectively. The suffix “–CO” denotes the corresponding constant-temperature oven control.

The influence of precursor structure was first reflected in the visual appearance of the products. As shown in Figure 1a, the aqueous dispersions of C-CDs, H-CDs, and P-CDs displayed yellow-green, yellow-brown, and reddish-brown colors under natural light, respectively. A similar color-deepening trend was observed for the corresponding freeze-dried powders in Figure 1b. These color differences indicate that the electronic structures and visible-light absorption abilities of the CDs were significantly affected by the functional precursors. C-CDs showed the lightest color, which can be attributed to the aliphatic structure of cysteine and its limited ability to promote extended conjugated-domain growth. H-CDs exhibited a deeper color, suggesting that the imidazole ring in histidine enhanced the formation of nitrogen-containing conjugated structures. P-CDs showed the darkest reddish-brown color, consistent with the strong ability of p-phenylenediamine to extend aromatic conjugation and increase visible-light absorption.

The photoluminescence behavior further confirmed the precursor-dependent regulation of the CDs. As shown in Figure 1e–g, the emission spectra of Cn-CDs, Hn-CDs, and Pn-CDs under 480 nm excitation varied significantly with the type and amount of functional precursor. A common excitation wavelength of 480 nm was selected for the precursor-loading series because all three sample series exhibited measurable excitation responses at this wavelength, allowing their long-wavelength emission profiles to be compared under identical excitation conditions. This result demonstrates that the functional precursor not only affects CD formation but also modulates the emissive states. For cysteine-modified CDs, the photoluminescence quantum yield (PLQY) first increased and then decreased with increasing cysteine dosage, as shown in Figure 1c. This trend suggests that cysteine plays a dual role during carbonization. At an appropriate dosage, cysteine can provide reactive amino and thiol groups, increase nucleation sites, and passivate surface defects at the carbon-core edges. These effects reduce non-radiative recombination and improve radiative transition efficiency. Meanwhile, Figure 1d shows that cysteine addition also increased the product yield compared with the system without cysteine, indicating that cysteine promoted the conversion of carbon sources into solid fluorescent products.

However, excessive cysteine caused a decline in PLQY. This phenomenon can be attributed to over-crosslinking-induced quenching. When the cysteine content is too high, abundant reactive groups may accelerate intermolecular dehydration and condensation, producing a dense crosslinked network. Such a network can shorten the distance between emissive domains, enhance chromophore-chromophore interactions, and increase non-radiative energy dissipation. Therefore, an optimized cysteine dosage is necessary to balance surface passivation and structural over-condensation.

The optimized PLQYs and product yields of the three CDs are summarized in Table S1. C-CDs, H-CDs, and P-CDs showed PLQYs of 29.62%, 50.98%, and 40.78%, respectively. Among them, H-CDs exhibited the highest PLQY, indicating that histidine is particularly effective in improving fluorescence efficiency in the solvent-free lignin-based system. This enhancement is mainly associated with the imidazole ring of histidine, which facilitates nitrogen incorporation and graphitic nitrogen formation during carbonization. Graphitic nitrogen can regulate the electronic density of sp^2^ carbon domains, enhance π-electron delocalization, and suppress non-radiative recombination. As a result, the radiative recombination efficiency of H-CDs is significantly improved.

Although P-CDs did not show the highest PLQY, they exhibited the most pronounced long-wavelength emission. The emission maximum of P-CDs shifted to approximately 573 nm, indicating a significant red shift compared with the other samples. This red-shifted emission can be attributed to the rigid aromatic skeleton and strong electron-donating ability of p-phenylenediamine. During carbonization, p-phenylenediamine can interact with lignin-derived aromatic fragments through π-π interactions and participate in the construction of extended conjugated domains. In addition, the amino groups of p-phenylenediamine may interact with oxygen-containing groups in lignin, generating charge-transfer-related emissive states. Both conjugation extension and charge-transfer interactions reduce the effective energy gap of the CDs, resulting in emission at longer wavelengths [39].

The product yields also showed a clear precursor-dependent trend. As listed in Table S1, C-CDs and H-CDs exhibited relatively high yields of 31.80% and 33.13%, respectively, whereas P-CDs showed a much lower yield of 11.18%. This difference indicates that high fluorescence performance and high product yield are controlled by related but not identical factors. Cysteine and histidine can participate in condensation reactions and stabilize carbonized intermediates, while the foaming behavior promoted by lysine provides spatial confinement for CD formation. In contrast, p-phenylenediamine lacks easily cleavable gas-generating groups and tends to undergo premature carbonization or aromatic stacking. These processes weaken the foaming-assisted spatial confinement effect and reduce the conversion efficiency into well-dispersed CDs. Therefore, the photoluminescence properties and synthetic efficiency of lignin-derived CDs are jointly governed by the molecular structure of the functional precursor and the confined carbonization environment.

### 3.2. Morphology, Particle Size, Optical Absorption, and Emission Characteristics

**Figure 2:**
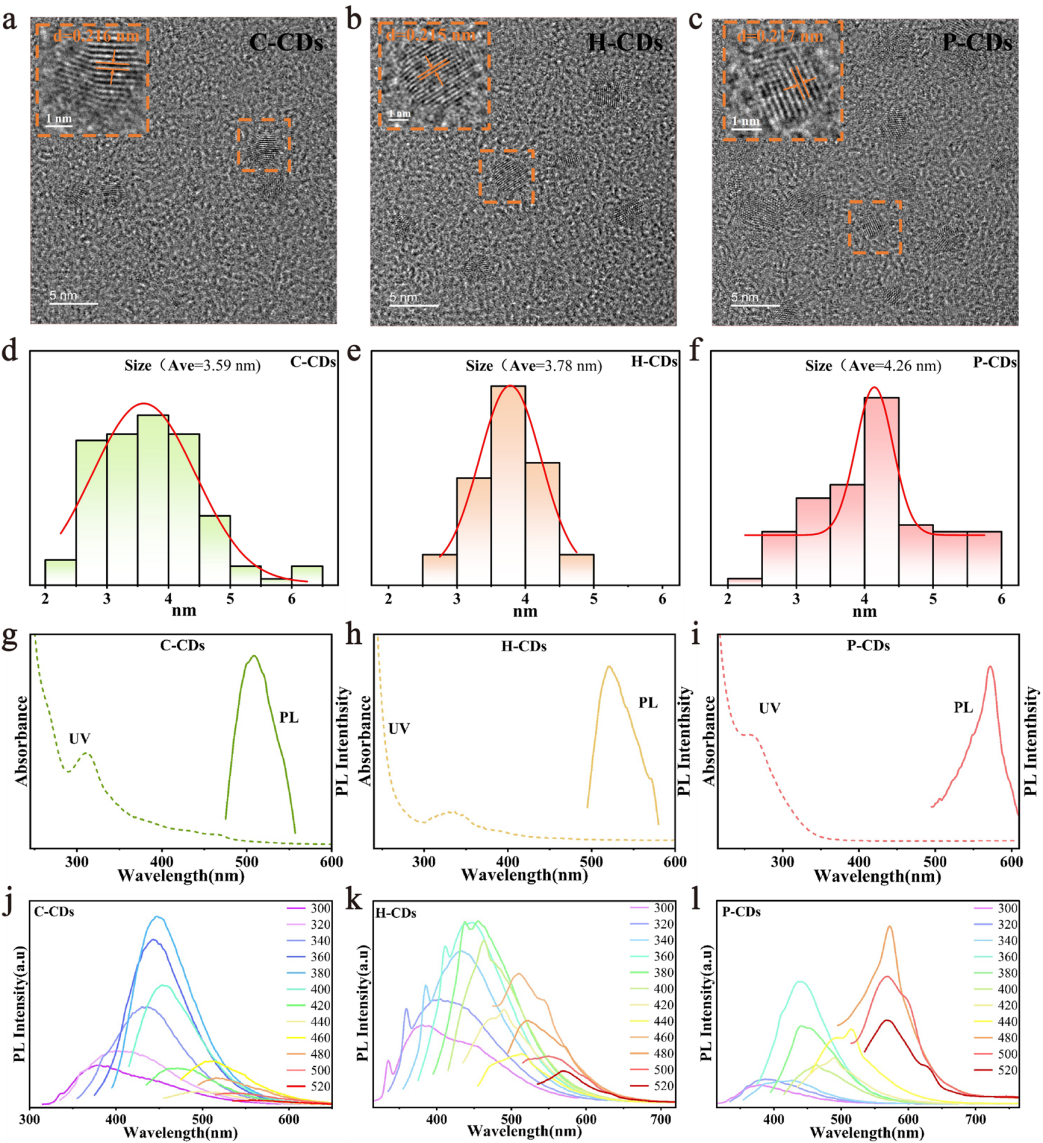
HR-TEM images of (**a**) C-CDs, (**b**) H-CDs, and (**c**) P-CDs. Particle size distribution histograms of (**d**) C-CDs, (**e**) H-CDs, and (**f**) P-CDs. (**g**–**i**) UV-Vis absorption and PL spectra of C-CDs, H-CDs, and P-CDs; (**j**–**l**) PL spectra of C-CDs, H-CDs, and P-CDs at different excitation wavelengths.

The morphology and microstructure of the precursor-engineered CDs were characterized by high-resolution transmission electron microscopy. As shown in Figure 2a–c and Figure S1, C-CDs, H-CDs, and P-CDs all displayed nearly spherical nanoparticles with good dispersibility. No obvious aggregation or large carbonized residues were observed, indicating that the solvent-free spatially confined carbonization strategy, followed by centrifugation, dialysis, filtration, and freeze-drying, produced well-dispersed nanoscale CDs. The uniform morphology also suggests that the locally confined reaction environment effectively restricted uncontrolled growth of carbonized intermediates.

High-resolution TEM images revealed clear lattice fringes in the three CDs (Figure 2a–c). The interplanar spacing was approximately 0.216 nm, which can be assigned to graphitic carbon domains. This result confirms that lignin aromatic units underwent condensation, aromatization, and partial graphitization during thermal treatment. Although the overall carbon structure remained partially amorphous, the presence of graphitic domains indicates the formation of sp^2^ carbon-core structures, which are essential for photoluminescence generation and electronic transitions.

A comparison among the three samples shows that their local structural order differed. C-CDs contained both amorphous regions and graphitic lattice domains, suggesting a relatively disordered carbon-core structure. This may be caused by the aliphatic nature and thermal decomposition behavior of cysteine, which can disturb the ordered stacking of lignin-derived aromatic fragments. In contrast, H-CDs and P-CDs exhibited clearer and more extended lattice fringes, indicating a higher degree of local graphitization. For H-CDs, the imidazole ring of histidine may promote nitrogen incorporation and ordered carbon-core growth. For P-CDs, the aromatic skeleton of p-phenylenediamine can enhance π-π interactions with lignin-derived aromatic units and facilitate conjugated-domain extension. These morphological observations are consistent with the precursor-dependent optical behavior discussed above.

The particle size distributions are shown in Figure 2d–f. C-CDs, H-CDs, and P-CDs had average diameters of approximately 3.59, 3.78, and 4.26 nm, respectively, with most particles distributed within 2–6 nm. These sizes are within the typical range of carbon quantum dots. The slight increase in average particle size from C-CDs to P-CDs suggests that aromatic and heterocyclic precursors may promote the growth of carbonized domains. In particular, P-CDs showed the largest average size, which may be attributed to stronger aromatic condensation and π-π stacking induced by p-phenylenediamine. The relatively narrow size distribution of H-CDs suggests that histidine helped regulate nucleation and growth more uniformly in the confined carbonization environment. By contrast, the broader size distribution of C-CDs indicates that cysteine may lead to less uniform carbon-core evolution because of its easier thermal decomposition.

The UV-vis absorption spectra and PL spectra further provide information on the electronic structures of the CDs. As shown in Figure 2g–i, all three CDs exhibited an absorption band around 280 nm. This band is generally assigned to the π→π* transition of aromatic C=C bonds, indicating the formation of conjugated sp^2^ domains. In addition, shoulder absorption in the 300–350 nm region was observed, corresponding to n→π* transitions of C=O or C=N groups [4]. The presence of these absorption features indicates that the CDs contained both aromatic carbon cores and surface oxygen/nitrogen-containing functional groups.

Although the UV-vis absorption features of the three CDs were similar, their PL emission behaviors were markedly different. This indicates that the fluorescence differences cannot be explained solely by the presence of basic sp^2^ carbon frameworks. Instead, the emissive behavior is strongly influenced by surface states, nitrogen configurations, sulfur incorporation, and conjugated-domain distributions. H-CDs showed high fluorescence efficiency, whereas P-CDs showed a distinct red-shifted emission. These differences correspond well to the molecular structures of histidine and p-phenylenediamine, respectively.

As shown in Figure 2j–l, the three CDs exhibited excitation-dependent emission behavior. With increasing excitation wavelength, the emission peaks shifted to different degrees. Such excitation dependence is a common feature of CDs and usually reflects the existence of multiple emissive centers or heterogeneous energy levels [40]. In the present system, these emissive centers may include carbon-core-related states, surface defect states, heteroatom-induced states, and molecular or conjugated structures derived from the functional precursors. The excitation-dependent behavior therefore supports the conclusion that the photoluminescence of these lignin-derived CDs results from the combined contribution of carbon-core states and precursor-regulated surface or molecular states.

For C-CDs, the relatively disordered carbon-core structure and sulfur-containing surface sites may create diverse surface defect states, resulting in moderate fluorescence efficiency. For H-CDs, the enhanced graphitic nitrogen content and more ordered carbon-core structure contribute to efficient radiative recombination. For P-CDs, the extended aromatic conjugation and charge-transfer-related structures shift the emission toward longer wavelengths. Therefore, the morphology, particle size, UV-vis absorption, and PL emission results collectively demonstrate that precursor molecular structure plays a central role in regulating both the physical structure and optical properties of lignin-derived CDs.

### 3.3. Surface Chemical Composition and Structural Origin of Tunable Photoluminescence

**Figure 3:**
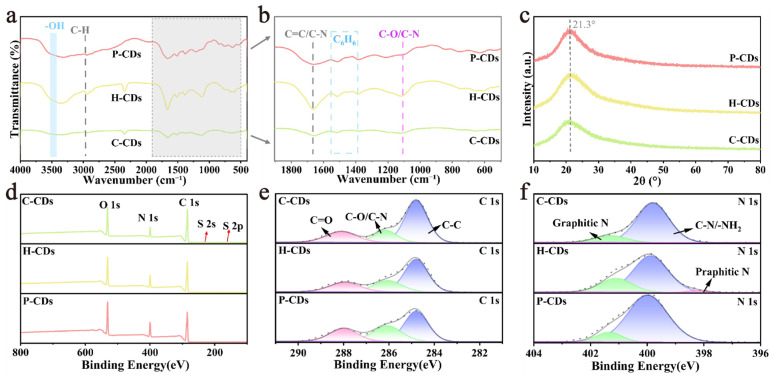
FTIR spectra of (**a**) C-CDs, H-CDs, and P-CDs and (**b**) enlarged FTIR spectra; (**c**) XRD patterns; (**d**) XPS survey spectra; high-resolution fitted spectra of (**e**) C 1s and (**f**) N 1s.

To further reveal the structural origin of the precursor-dependent photoluminescence, FTIR, XRD, and XPS analyses were performed. FTIR spectroscopy was first used to identify the surface functional groups of the CDs. As shown in Figure 3a,b, all three CDs exhibited broad absorption bands at 3400–3500 cm^−1^, which are assigned to O-H and N-H stretching vibrations. These bands indicate that the CD surfaces contained abundant hydroxyl and amino groups. Such hydrophilic groups are important for maintaining good water dispersibility and can also act as active sites for metal-ion coordination.

The absorption near 2920 cm^−1^ corresponds to aliphatic C-H stretching vibrations, indicating the presence of residual aliphatic structures or surface alkyl fragments. The peak around 1650 cm^−1^ can be attributed to C=C or C=N stretching vibrations in aromatic or conjugated structures, confirming the formation of conjugated carbon domains. The absorption around 1100 cm^−1^ is associated with C-O or C-N vibrations, suggesting that oxygen- and nitrogen-containing groups were retained or generated during carbonization. The similarity of the major FTIR peaks indicates that the three CDs possessed comparable types of surface functional groups. However, differences in peak intensity and chemical-state distribution, as further confirmed by XPS, indicate that the relative abundance and bonding configurations of these groups varied with the functional precursor.

The carbon structural order was investigated by XRD. As shown in Figure 3c, all CDs exhibited broad diffraction peaks centered at approximately 21.3°. The broad nature of the diffraction peaks indicates that the CDs were mainly composed of amorphous or low-graphitized carbon structures. This result is consistent with the TEM observation that the CDs contained short-range ordered graphitic domains but lacked long-range crystalline order. Compared with C-CDs, H-CDs and P-CDs showed slightly sharper diffraction peaks, suggesting a modest increase in local carbon ordering. This trend agrees with the clearer lattice fringes observed in Figure 2a–c and supports the role of histidine and p-phenylenediamine in promoting carbon-core organization.

XPS analysis provided detailed information on elemental composition and chemical bonding states. As shown in Figure 3d, C, N, and O were detected in all samples, while S was detected only in C-CDs. This confirms that nitrogen and oxygen were common elements in all CDs, whereas sulfur was specifically introduced by cysteine. The high-resolution C 1s spectra in Figure 3e were deconvoluted into C-C/C=C, C-O/C-N, and C=O/O-C=O components. According to Table S2, C-C/C=C was the dominant carbon species in all CDs, confirming that sp^2^ carbon frameworks were successfully constructed. H-CDs showed a relatively higher C-C/C=C content than the other samples, indicating a higher degree of carbon-core conjugation. This result is consistent with the high PLQY and improved local graphitization of H-CDs.

The N 1s spectra shown in Figure 3f reveal key differences among the three CDs. Nitrogen species mainly existed as graphitic nitrogen, amino-related nitrogen, and pyridinic nitrogen. H-CDs contained a relatively high proportion of graphitic nitrogen, accounting for more than one quarter of the total nitrogen species. Graphitic nitrogen is incorporated into the carbon framework and can effectively modulate the electronic structure of sp^2^ domains. Its presence enhances π-electron delocalization, improves electronic transition pathways, and reduces non-radiative recombination. Therefore, the high graphitic nitrogen content provides direct structural evidence for the superior PLQY of H-CDs.

In contrast, P-CDs showed a higher proportion of -NH_2_-related nitrogen species. This result indicates that p-phenylenediamine introduced amino-rich surface structures during carbonization. These amino groups may play two roles. First, they can contribute to surface-state emission by modifying the electronic environment of the CDs. Second, they can serve as coordination sites for metal ions, especially Ag^+^. The red-shifted emission of P-CDs is therefore associated not only with enlarged aromatic conjugation but also with amino-modified surface states and possible charge-transfer structures.

For C-CDs, the high-resolution S 2p spectrum in Figure S3 confirmed the presence of C-S bonds. This demonstrates that sulfur from cysteine was successfully incorporated into the CDs, although the sulfur content was relatively low. The absence of strong sulfur-related absorption in the FTIR spectra can be explained by the low sulfur content and overlap with other vibrational bands. Even at low content, sulfur-containing sites can significantly affect coordination behavior because sulfur atoms have strong affinity toward certain metal ions and can alter the local electronic environment of the CD surface.

The O 1s spectra in Figure S2 further confirmed the presence of oxygen-containing groups such as C=O and C-O. These groups are important for maintaining aqueous stability and providing metal-ion binding sites. The coexistence of carbonyl, hydroxyl, amino, and sulfur-containing groups creates a chemically rich surface environment for fluorescence sensing.

Overall, the FTIR, XRD, and XPS results show that the three CDs shared similar basic carbon skeletons but differed in local graphitization, nitrogen configuration, sulfur incorporation, and surface amino-group distribution. These structural differences explain the distinct optical behaviors of the CDs. H-CDs achieved the highest PLQY because histidine promoted graphitic nitrogen formation and carbon-core conjugation. P-CDs showed red-shifted emission because p-phenylenediamine extended π-conjugated domains and introduced amino-rich surface states. C-CDs showed moderate fluorescence and Fe^3+^-responsive surface chemistry because cysteine introduced sulfur-containing coordination sites. Thus, the structural characterization results establish a direct connection between precursor structure and photoluminescence regulation. These surface chemical features provide potential metal-binding sites, while their actual involvement in the sensing process is examined below by comparing the pristine and metal-treated CDs.

### 3.4. Environmental Stability and Selective Fluorescence Sensing of Metal Ions

**Figure 4:**
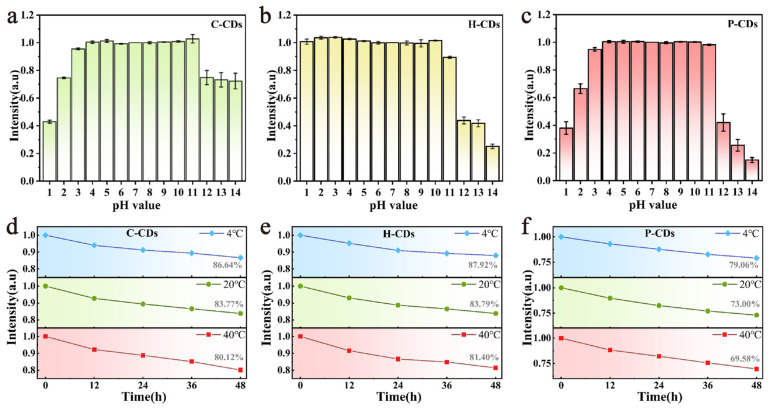
(**a**–**c**) pH-dependent PL intensity and (**d**–**f**) PL stability under visible light irradiation at different temperatures of C-CDs, H-CDs, and P-CDs. The data in panels (**a**–**c**) are presented as the mean ± SD of three replicate measurements (*n* = 3), and the error bars represent SD. Panels (**d**–**f**) show representative stability measurements.

The environmental stability of fluorescent probes is crucial for practical sensing applications. Therefore, the fluorescence responses of C-CDs, H-CDs, and P-CDs were evaluated under different pH and thermal/light conditions. As shown in Figure 4a–c, C-CDs and P-CDs maintained relatively stable fluorescence intensity in the pH range of 3–11. However, their fluorescence was significantly quenched under strongly acidic or strongly alkaline conditions. This behavior suggests that the emissive states of C-CDs and P-CDs are stable under neutral and weakly acidic or alkaline conditions but are sensitive to extreme pH environments. Under strong acid or base, surface functional groups such as hydroxyl, carboxyl, amino, or carbonyl groups may undergo protonation or deprotonation, altering the surface electronic structure and increasing non-radiative recombination pathways [41].

H-CDs displayed better pH tolerance than the other two samples. Its fluorescence intensity remained stable from pH 1 to 11 and decreased significantly only under strongly alkaline conditions. This broad pH stability can be attributed to the relatively high graphitic nitrogen content of H-CDs. Unlike surface amino or carboxyl groups, graphitic nitrogen is embedded in the carbon framework and is less susceptible to protonation or deprotonation. Therefore, the electronic structure and emissive states of H-CDs are more stable over a wide pH range. This result is consistent with the XPS analysis, which shows that histidine promotes graphitic nitrogen formation.

The thermal and light stability results are shown in Figure 4d–f. Under visible-light irradiation, the fluorescence intensity of all CDs gradually decreased with increasing irradiation time, and the fluorescence decay became more pronounced at higher temperatures. This trend can be explained by thermally activated non-radiative relaxation [42]. As the temperature increases, molecular vibration and rotation become more intense, leading to enhanced energy dissipation through non-radiative pathways. Among the three samples, P-CDs showed the most obvious attenuation at 40 °C after 48 h, retaining approximately 70% of the initial fluorescence intensity. The lower thermal stability of P-CDs may be related to its higher content of surface amino groups. These amino groups are sensitive to hydrogen-bond rearrangement and surface-state fluctuation under thermal disturbance, which can affect the emissive states and accelerate fluorescence decay.

Despite fluorescence attenuation under high temperature and prolonged irradiation, the three CDs maintained acceptable fluorescence stability under conditions relevant to aqueous environmental sensing. Their stability in neutral and weakly acidic or alkaline media provides a basis for subsequent metal-ion detection experiments.

**Figure 5:**
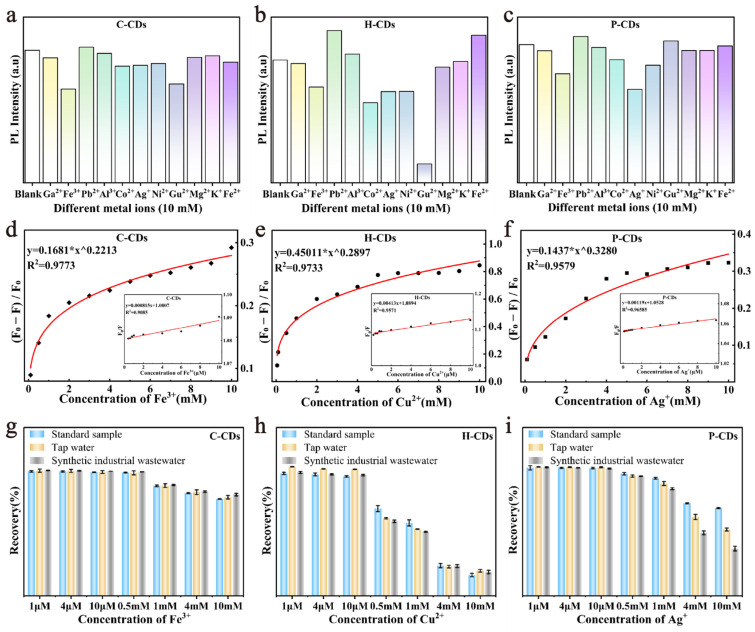
Fluorescence intensity changes of (**a**) C-CDs, (**b**) H-CDs, and (**c**) P-CDs in the presence of different metal ions (10 mM). Fluorescence response and linear fitting curves of different carbon dots toward target metal ions. (**d**) Fluorescence response and low-concentration linear fitting of C-CDs toward Fe^3+^. (**e**) Fluorescence response and low-concentration linear fitting of H-CDs toward Cu^2+^. (**f**) Fluorescence response and low-concentration linear fitting of P-CDs toward Ag^+^. The insets show the low-concentration Stern–Volmer linear fits Comparison of fluorescence response of (**g**) C-CDs, (**h**) H-CDs, and (**i**) P-CDs in different water matrices. The data in panels (**g**–**i**) are presented as the mean ± SD of three replicate measurements (*n* = 3), and the error bars represent SD. Panels (**a**–**f**) show representative measurements.

The metal-ion selectivity of the CDs was investigated by adding different metal ions and recording fluorescence intensity changes. As shown in Figure 5a–c, the three CDs exhibited clearly different fluorescence responses toward metal ions, demonstrating that their ion-recognition behavior was strongly influenced by precursor-induced surface chemistry. C-CDs showed a selective response toward Fe^3+^. This selectivity can be attributed to sulfur- and oxygen-containing functional groups introduced by cysteine. Fe^3+^ has strong affinity for oxygen-containing groups such as hydroxyl and carbonyl groups, and sulfur-containing sites may further enhance complexation. The observed response is consistent with the interaction of Fe^3+^ with oxygen- and sulfur-containing surface sites. The nature of the quenching process is discussed below using the fluorescence-lifetime and XPS results.

H-CDs exhibited the strongest fluorescence response toward Cu^2+^. In the presence of high Cu^2+^ concentration, the fluorescence intensity of H-CDs decreased to approximately 15% of its initial value, indicating highly efficient quenching. This strong response is mainly attributed to the imidazole-derived nitrogen sites introduced by histidine. Imidazole nitrogen atoms possess strong coordination ability toward Cu^2+^, enabling the formation of stable surface complexes. The strong response is consistent with the interaction of Cu^2+^ with histidine-derived nitrogen sites. However, the steady-state fluorescence response alone does not establish the corresponding excited-state pathway. In addition, the high graphitic nitrogen content of H-CDs may facilitate coupling between the carbon core and surface interaction sites, further improving quenching efficiency and sensing sensitivity.

P-CDs displayed a relatively selective response toward Ag^+^. The amino-rich surface derived from p-phenylenediamine provides suitable binding sites for Ag^+^ coordination. At higher Ag^+^ concentrations, solution turbidity was observed, suggesting possible interparticle interactions. In the absence of direct particle-size measurements, aggregation is considered a possible secondary contribution rather than a confirmed mechanism [43].

These results show that selective ion recognition can be achieved by regulating the surface coordination environment of lignin-derived CDs through precursor molecular engineering. Cysteine favors Fe^3+^ recognition, histidine favors Cu^2+^ recognition, and p-phenylenediamine favors Ag^+^ recognition. The sensing behavior therefore follows the structural features of the functional precursors.

### 3.5. Quantitative Detection, Practical Water-Sample Analysis, and Structure–Property Relationship

After confirming the selective fluorescence responses, quantitative sensing experiments were conducted by adding different concentrations of the corresponding target ions. As shown in Figure 5d–f, the fluorescence intensities of C-CDs, H-CDs, and P-CDs decreased progressively with increasing concentrations of Fe^3+^, Cu^2+^, and Ag^+^, respectively. The continuous decrease in fluorescence intensity indicates that the target ions effectively interact with the CD surfaces and induce concentration-dependent quenching.

At relatively high ion concentrations, the fluorescence intensity decreased sharply. This strong quenching can be attributed to the rapid occupation of surface coordination sites and enhanced electron-transfer or aggregation-related processes. At low concentrations, the fluorescence intensity exhibited an approximately linear relationship with ion concentration, allowing quantitative detection. The corresponding linear calibration curves are shown in inserts of Figure 5d–f, and the analytical parameters are summarized in Table S3.

For C-CDs, a linear response toward Fe^3+^ was obtained in the range of 0.4–10 μM, with a correlation coefficient of 0.9085 and a limit of detection (LOD) of 0.26 μM. Although the linearity was lower than those of H-CDs and P-CDs, the result still confirms the feasibility of cysteine-modified lignin-derived CDs for Fe^3+^ detection. The moderate sensitivity of C-CDs may be related to the relatively low sulfur content and the heterogeneous distribution of surface binding sites.

For H-CDs, a good linear response toward Cu^2+^ was observed in the range of 0.2–10 μM, with a correlation coefficient of 0.9571 and an LOD as low as 0.05 μM. This was the lowest detection limit among the three systems, indicating that H-CDs possessed the highest sensing sensitivity. The excellent Cu^2+^ detection performance arises from the combination of high PLQY, stable emissive structure, and strong Cu^2+^ coordination ability of histidine-derived nitrogen sites. A high initial fluorescence intensity provides a larger signal window, while the specific Cu^2+^ binding sites ensure efficient fluorescence quenching [44].

For P-CDs, the fluorescence intensity showed a linear response to Ag^+^ over the range of 0.4–10 μM, with a correlation coefficient of 0.9659 and an LOD of 0.11 μM. The relatively good linearity indicates that Ag^+^-induced quenching occurred in a concentration-dependent manner. The amino-rich surface of P-CDs provides potential Ag^+^ interaction sites, while interparticle interactions may provide an additional contribution at elevated Ag^+^ concentrations. The low-concentration data showed approximately linear Stern–Volmer relationships. The apparent Stern–Volmer constants were 8.15 × 10^2^, 4.13 × 10^3^, and 1.19 × 10^3^ M^−1^ for C-CDs/Fe^3+^, H-CDs/Cu^2+^, and P-CDs/Ag^+^, respectively. The non-unity intercepts and deviations from linearity over the broader millimolar concentration range indicate non-ideal Stern–Volmer behavior, which may reflect heterogeneous surface sites and progressive occupation of the available interaction sites. The Stern–Volmer results were therefore not used alone to distinguish static from dynamic quenching. The corresponding linear ranges, coefficients of determination, apparent Stern–Volmer constants, and detection limits are summarized in Table S3.

The mixed-ion experiments revealed probe-dependent matrix effects, as shown in Figure S8 and summarized quantitatively in Table S6. For C-CDs, the mixed-ion background alone caused a 4.5% decrease in fluorescence intensity, while the net Fe^3+^-induced quenching increased from 25.3% to 40.6%. For H-CDs, the mixed-ion background decreased the baseline fluorescence by 22.1%, whereas the net Cu^2+^-induced quenching changed from 87.3% to 83.8%. For P-CDs, the mixed-ion background caused a 5.3% decrease, while the Ag^+^-induced quenching changed from 35.2% to 39.3%. Thus, the target-ion responses remained distinguishable under the tested competitive ionic conditions, although appreciable probe-dependent matrix effects were observed.

The practical applicability of the CDs was further evaluated in deionized water, tap water, and simulated wastewater. As shown in Figure 5g–i, C-CDs, H-CDs, and P-CDs retained effective fluorescence responses toward their target ions in different water matrices. These results indicate that the target-ion responses remained detectable in the tested water matrices, although their magnitudes were affected by the sample composition. For C-CDs and H-CDs, the fluorescence quenching efficiencies in tap water and simulated wastewater were close to those in deionized water, indicating that the Fe^3+^ and Cu^2+^ responses were retained in the tested matrices, although complete absence of matrix interference cannot be assumed. These results demonstrate that the fluorescence responses were retained in the tested water matrices. However, because spike recoveries against independently known concentrations were not determined, these experiments should be regarded as matrix-tolerance tests rather than full analytical validation in real samples.

In contrast, P-CDs showed slightly stronger fluorescence quenching in tap water and simulated wastewater, especially at higher Ag^+^ concentrations. This phenomenon may be caused by the higher ionic strength of complex water matrices. Coexisting ions can compress the electrical double layer on the CD surface, reduce electrostatic repulsion between particles, and promote Ag^+^-mediated aggregation [45]. In addition, some ions or components in complex water samples may participate in cooperative coordination, further enhancing the interaction between Ag^+^ and amino-rich P-CD surfaces. Although matrix effects influenced the response of P-CDs to some extent, the fluorescence quenching remained clear, indicating that P-CDs still possess practical sensing potential.

### 3.6. Metal-Ion Coordination and Fluorescence-Quenching Mechanism

To clarify the fluorescence-quenching mechanism and distinguish static coordination from dynamic collisional quenching, the concentration-dependent fluorescence responses, time-resolved fluorescence decay curves, and XPS spectra before and after metal-ion addition were analyzed collectively. The XPS comparison focused on changes in heteroatom-related surface components and on the appearance of metal-associated signals. The C–C/C=C component at 284.8 eV was used only for charge correction and was therefore not interpreted as independent evidence that the carbon core remained unchanged.

For C-CDs/Fe^3+^, the C–O/C–N component shifted from approximately 286.15 to 286.68 eV. The O 1s components originally located at approximately 531.45 and 533.20 eV were redistributed after Fe^3+^ addition, accompanied by changes in the S 2p component distribution. Characteristic Fe 2p features were observed at approximately 711.68 and 724.88 eV. These results support the participation of oxygen- and cysteine-derived sulfur-containing sites in Fe^3+^ coordination. For H-CDs/Cu^2+^, the principal N 1s component shifted from approximately 399.90 to 399.58 eV, and the O 1s envelope also underwent substantial redistribution. The appearance of Cu 2p features at approximately 932.98 and 952.78 eV further confirmed the association of Cu^2+^ with H-CDs. These changes are consistent with coordination involving histidine-derived nitrogen sites and oxygen-containing surface groups. For P-CDs/Ag^+^, changes were observed in both the N 1s and O 1s regions. In addition, a Ag 3d doublet was detected at approximately 367.48 and 373.58 eV, with a spin–orbit separation of approximately 6.10 eV. These results support Ag^+^ coordination involving the amino-rich nitrogen sites and oxygen-containing groups on the P-CD surface. The complete charge-corrected spectra of the three metal-treated systems and the representative binding-energy changes are provided in Figures S5–S7 and Table S5, respectively.

The fluorescence decay curves were adequately described using a biexponential model, and the corresponding decay curves and fitting parameters are presented in Figure S4 and Table S4, respectively. The intensity-weighted average lifetime changed only from 4.741 to 4.794 ns for C-CDs/Fe^3+^ and from 6.634 to 6.582 ns for P-CDs/Ag^+^. The substantial fluorescence-intensity decrease without appreciable lifetime shortening is consistent with a predominantly static, coordination-mediated quenching contribution in these two systems. For H-CDs/Cu^2+^, the average lifetime increased from 4.645 to 5.858 ns, while the contribution of the long-lived component increased from 61.07% to 69.48%. This behavior is inconsistent with a dominant dynamic collisional-quenching pathway and suggests that Cu^2+^ coordination preferentially suppresses short-lived emissive sites, thereby redistributing the residual emission toward longer-lived surface states. Therefore, the H-CDs/Cu^2+^ response cannot be described as simple static quenching solely on the basis of the lifetime data.

Taken together, none of the three systems exhibited the lifetime shortening expected for a dominant dynamic collisional process. The XPS and lifetime results therefore support a major contribution from metal–surface coordination rather than a dominant dynamic collisional-quenching pathway. For C-CDs/Fe^3+^ and P-CDs/Ag^+^, the nearly unchanged average lifetimes are consistent with predominantly static coordination-mediated quenching, whereas H-CDs/Cu^2+^ exhibits more complex site-selective behavior. A possible aggregation-assisted contribution may occur at elevated metal-ion concentrations, particularly in the P-CDs/Ag^+^ system, but aggregation is treated as a secondary possibility rather than as a conclusively demonstrated mechanism. Because direct particle-size and electrokinetic measurements were not available, metal-induced aggregation could not be evaluated conclusively. The observed turbidity is therefore discussed only as qualitative evidence of a possible secondary contribution at elevated Ag^+^ concentrations. Direct measurements of hydrodynamic size and zeta potential as functions of metal-ion concentration would be required to confirm coordination-induced aggregation and identify any saturation behavior [46,47].

### 3.7. Precursor–Structure–Property Relationship

Based on the above results, a precursor–structure–property relationship can be established for the solvent-free lignin-derived CDs. The common solvent-free spatially confined carbonization system provides a favorable reaction environment for CD formation. Lysine contributes nitrogen and promotes foaming-assisted confinement, while oxalic acid facilitates dehydration and carbonization. Within this framework, the functional precursor determines the heteroatom configuration, surface functional groups, conjugated-domain structure, and ion-recognition behavior.

Cysteine participates in condensation reactions and introduces sulfur-containing coordination sites, leading to Fe^3+^-selective fluorescence response. Histidine promotes graphitic nitrogen formation through its imidazole ring, increasing carbon-core conjugation and improving PLQY. As a result, H-CDs combine high emission efficiency, broad pH stability, and strong Cu^2+^ coordination ability, achieving sensitive Cu^2+^ detection. p-Phenylenediamine extends π-conjugated domains through its rigid aromatic skeleton and introduces amino-rich surface sites, resulting in red-shifted emission and Ag^+^-selective response.

Therefore, the photoluminescence and sensing performances of lignin-derived CDs are jointly governed by carbon-core ordering, heteroatom configuration, surface-state distribution, and coordination-site construction. This study demonstrates that precursor molecular engineering is an effective strategy for simultaneously regulating fluorescence efficiency, emission wavelength, and ion-recognition selectivity in biomass-derived CDs.

## 4. Conclusions

In this study, lignin-derived carbon dots with tunable photoluminescence and selective metal-ion sensing capability were successfully prepared through a solvent-free precursor–structure–engineering strategy. The results confirm that precursor molecular structure plays a decisive role in determining the carbon-core ordering, heteroatom configuration, surface-state distribution, and coordination-site construction of lignin-derived CDs. Histidine was the most effective precursor for improving fluorescence efficiency, as its imidazole structure promoted graphitic nitrogen formation and enhanced carbon-core conjugation, leading to the highest photoluminescence quantum yield of 50.98% and improved pH stability. p-Phenylenediamine was more favorable for inducing long-wavelength emission because its rigid aromatic structure extended π-conjugated domains and generated charge-transfer-related emissive states, resulting in a red-shifted emission at approximately 573 nm, although its tendency toward aromatic stacking reduced the product yield. Cysteine contributed to surface passivation and sulfur-containing site formation, thereby improving product formation and providing coordination sites for Fe^3+^ recognition, but its aliphatic structure limited carbon-core ordering and fluorescence efficiency. The ion-sensing results further demonstrate that precursor-induced surface chemistry determines metal-ion selectivity: C-CDs, H-CDs, and P-CDs selectively responded to Fe^3+^, Cu^2+^, and Ag^+^, respectively, with detection limits of 0.26, 0.05, and 0.11 μM. Their responses remained detectable in tap water and simulated wastewater, although probe-dependent matrix effects were observed. The quenching behavior was inconsistent with a dominant dynamic collisional process and was most consistent with a major contribution from metal–surface complexation. The nearly unchanged lifetimes of C-CDs/Fe^3+^ and P-CDs/Ag^+^ are consistent with predominantly static quenching, whereas the lifetime redistribution observed for H-CDs/Cu^2+^ indicates more complex site-selective coordination behavior. Possible aggregation at elevated metal-ion concentrations remains to be evaluated by direct colloidal measurements. Overall, this work establishes a precursor–structure–photoluminescence–sensing relationship for lignin-derived CDs and demonstrates that solvent-free precursor engineering is an effective strategy for designing biomass-derived fluorescent nanomaterials with tunable emission and targeted ion-recognition functions.

## Data Availability

Data is contained within the article or Supplementary Material.

## Supplementary Materials

The following supporting information can be downloaded at: https://www.mdpi.com/article/10.3390/nano16150931/s1. Figure S1. TEM images of (a) C-CDs, (b) H-CDs, and (c) P-CDs. Figure S2. XPS high-resolution fitted O 1s spectra of C-CDs, H-CDs, and P-CDs. Figure S3. XPS high-resolution fitted S 2p spectrum of C-CDs. Figure S4. Time-resolved photoluminescence decay curves and corresponding. Figure S5: Charge-corrected high-resolution XPS spectra and peak deconvolution of Fe^3+^-treated C-CDs: (a) C 1s, (b) O 1s, (c) N 1s, (d) S 2p, and (e) Fe 2p. Figure S6: Charge-corrected high-resolution XPS spectra and peak deconvolution of Cu^2+^-treated H-CDs: (a) C 1s, (b) O 1s, (c) N 1s, and (d) Cu 2p; Figure S7: Charge-corrected high-resolution XPS spectra and peak deconvolution of Ag^+^-treated P-CDs: (a) C 1s, (b) O 1s, (c) N 1s, and (d) Ag 3d; Figure S8. Normalized fluorescence emission spectra of (a) C-CDs/Fe^3+^, (b) H-CDs/Cu^2+^, and (c) P-CDs/Ag^+^ under single-ion and mixed-ion conditions. The spectra were recorded under 380 nm excitation, background-corrected, and normalized to the maximum fluorescence intensity of the corresponding blank CD dispersion. Table S1. Reaction conditions, fluorescence quantum yield, and product yield of different CDs. Table S2. Summary of selected XPS data for C-CDs, H-CDs, and P-CDs. Table S3. Analytical performance parameters of C-CDs, H-CDs, and P-CDs for metal ion detection. Table S4. Biexponential fitting parameters of the fluorescence decay curves of the pristine and target-metal-treated CDs. Table S5. Selected XPS binding energies of the pristine and target-metal-treated CDs. Table S6. Quantitative fluorescence responses of the three sensing systems under mixed-ion interference conditions.

## References

[1] Xu X., Ray R., Gu Y., Ploehn H.J., Gearheart L., Raker K., Scrivens W.A. (2004). Electrophoretic analysis and purification of fluorescent single-walled carbon nanotube fragments. J. Am. Chem. Soc..

[2] Sun Y.P., Zhou B., Lin Y., Wang W., Fernando K.A., Pathak P., Meziani M.J., Harruff B.A., Wang X., Wang H. (2006). Quantum-sized carbon dots for bright and colorful photoluminescence. J. Am. Chem. Soc..

[3] Baker S.N., Baker G.A. (2010). Luminescent carbon nanodots: Emergent nanolights. Angew. Chem. Int. Ed. Engl..

[4] Ding H., Li X.-H., Chen X.-B., Wei J.-S., Li X.-B., Xiong H.-M. (2020). Surface states of carbon dots and their influences on luminescence. J. Appl. Phys..

[5] Roy P., Chen P.-C., Periasamy A.P., Chen Y.-N., Chang H.-T. (2015). Photoluminescent carbon nanodots: Synthesis, physicochemical properties and analytical applications. Mater. Today.

[6] Sun X., Lei Y. (2017). Fluorescent carbon dots and their sensing applications. TrAC Trends Anal. Chem..

[7] Yoo D., Park Y., Cheon B., Park M.H. (2019). Carbon Dots as an Effective Fluorescent Sensing Platform for Metal Ion Detection. Nanoscale Res. Lett..

[8] Ding H., Yu S.B., Wei J.S., Xiong H.M. (2016). Full-Color Light-Emitting Carbon Dots with a Surface-State-Controlled Luminescence Mechanism. ACS Nano.

[9] Yuan F., Yuan T., Sui L., Wang Z., Xi Z., Li Y., Li X., Fan L., Tan Z., Chen A. (2018). Engineering triangular carbon quantum dots with unprecedented narrow bandwidth emission for multicolored LEDs. Nat. Commun..

[10] Dong Y., Pang H., Yang H.B., Guo C., Shao J., Chi Y., Li C.M., Yu T. (2013). Carbon-based dots co-doped with nitrogen and sulfur for high quantum yield and excitation-independent emission. Angew. Chem. Int. Ed. Engl..

[11] Ding H., Wei J.S., Xiong H.M. (2014). Nitrogen and sulfur co-doped carbon dots with strong blue luminescence. Nanoscale.

[12] Pan S., Li S., Deng S., Li X. (2026). A novel and effective N, S co-doped lignin based carbon dots inhibitor for cold rolled steel in 1.0 M HCl solution. Adv. Compos. Hybrid Mater..

[13] Barhum H., Alon T., Attrash M., Machnev A., Shishkin I., Ginzburg P. (2021). Multicolor Phenylenediamine Carbon Dots for Metal-Ion Detection with Picomolar Sensitivity. ACS Appl. Nano Mater..

[14] Li P., Xue S., Sun L., Zong X., An L., Qu D., Wang X., Sun Z. (2022). Formation and fluorescent mechanism of red emissive carbon dots from o-phenylenediamine and catechol system. Light Sci. Appl..

[15] Zeng Q., Feng T., Tao S., Zhu S., Yang B. (2021). Precursor-dependent structural diversity in luminescent carbonized polymer dots (CPDs): The nomenclature. Light Sci. Appl..

[16] Xia C., Zhu S., Feng T., Yang M., Yang B. (2019). Evolution and Synthesis of Carbon Dots: From Carbon Dots to Carbonized Polymer Dots. Adv. Sci..

[17] Kang C., Huang Y., Yang H., Yan X.F., Chen Z.P. (2020). A Review of Carbon Dots Produced from Biomass Wastes. Nanomaterials.

[18] Torres Landa S.D., Reddy Bogireddy N.K., Kaur I., Batra V., Agarwal V. (2022). Heavy metal ion detection using green precursor derived carbon dots. iScience.

[19] Ma B., Xiong F., Wang H., Qing Y., Chu F., Wu Y. (2023). Tailorable and scalable production of eco-friendly lignin micro-nanospheres and their application in functional superhydrophobic coating. Chem. Eng. J..

[20] Ma B., Xiong F., Wang H., Wen M., Yang J., Qing Y., Chu F., Wu Y. (2024). A gravity–inspired design for robust and photothermal superhydrophobic coating with dual–size lignin micro–nanospheres. J. Clean. Prod..

[21] Ma B., Wei Z., Chen L., Tu S., Qiu X. (2024). Superhydrophobic coatings with robustness and multifunctionality for wood protection by rod-like ZnO/lignin nanosphere composites design. Constr. Build. Mater..

[22] Vasile C., Baican M. (2023). Lignins as Promising Renewable Biopolymers and Bioactive Compounds for High-Performance Materials. Polymers.

[23] Wang R., Zhang S., Zhang J., Wang J., Bian H., Jin L., Zhang Y. (2024). State-of-the-art of lignin-derived carbon nanodots: Preparation, properties, and applications. Int. J. Biol. Macromol..

[24] Yin X., Zhang Z., Li F., Fu M., Qin T., Ji X., Wang Y., Wang Z., Sun S. (2025). Applications of Lignin-Dervied Carbon Quantum Dots: Current Status and Challenges. Exploration.

[25] Myint A.A., Rhim W.-K., Nam J.-M., Kim J., Lee Y.-W. (2018). Water-soluble, lignin-derived carbon dots with high fluorescent emissions and their applications in bioimaging. J. Ind. Eng. Chem..

[26] Lin S., Lai C., Huang Z., Liu W., Xiong L., Wu Y., Jin Y. (2023). Sustainable synthesis of lignin-derived carbon dots with visible pH response for Fe^3+^ detection and bioimaging. Spectrochim. Acta A Mol. Biomol. Spectrosc..

[27] Wu J., Li T., Qiu X., Qin Y., Chen L. (2023). γ-Valerolactone/H2O Binary Solvent for One-Pot Preparation and Functional Tailoring of Lignin-Based Carbon Dots. ACS Sustain. Chem. Eng..

[28] Li Y., Ren J., Sun R., Wang X. (2018). Fluorescent Lignin Carbon Dots for Reversible Responses to High-Valence Metal Ions and Its Bioapplications. J. Biomed. Nanotechnol..

[29] Wang R., Jiao L., Zhou X., Guo Z., Bian H., Dai H. (2021). Highly fluorescent graphene quantum dots from biorefinery waste for tri-channel sensitive detection of Fe^3+^ ions. J. Hazard. Mater..

[30] Zhang B., Liu Y., Ren M., Li W., Zhang X., Vajtai R., Ajayan P.M., Tour J.M., Wang L. (2019). Sustainable Synthesis of Bright Green Fluorescent Nitrogen-Doped Carbon Quantum Dots from Alkali Lignin. ChemSusChem.

[31] Kumari S., Sandhu N. (2026). Review on Tailored Carbon Dots for High-Performance Heavy Metal Ion Sensing in Aquatic Systems. Luminescence.

[32] Kanwal A., Bibi N., Hyder S., Muhammad A., Ren H., Liu J., Lei Z. (2022). Recent advances in green carbon dots (2015–2022): Synthesis, metal ion sensing, and biological applications. Beilstein J. Nanotechnol..

[33] Zhang Z., Chen X., Fang G., Wu J., Gao A. (2022). Self-carbonization synthesis of highly-bright red/near-infrared carbon dots by solvent-free method. J. Mater. Chem. C.

[34] Wang B., Lu S. (2022). The light of carbon dots: From mechanism to applications. Matter.

[35] Gan J., Chen L., Chen Z., Zhang J., Yu W., Huang C., Wu Y., Zhang K. (2023). Lignocellulosic Biomass-Based Carbon Dots: Synthesis Processes, Properties, and Applications. Small.

[36] Stagi L., Malfatti L., Caboi F., Innocenzi P. (2021). Thermal Induced Polymerization of l-Lysine forms Branched Particles with Blue Fluorescence. Macromol. Chem. Phys..

[37] Sun C., Zhang Y., Wang P., Yang Y., Wang Y., Xu J., Wang Y., Yu W.W. (2016). Synthesis of Nitrogen and Sulfur Co-doped Carbon Dots from Garlic for Selective Detection of Fe^3+^. Nanoscale Res. Lett..

[38] Lin S., Lin C., He M., Yuan R., Zhang Y., Zhou Y., Xiang W., Liang X. (2017). Solvatochromism of bright carbon dots with tunable long-wavelength emission from green to red and their application as solid-state materials for warm WLEDs. RSC Adv..

[39] Zhang Q., Wang R., Feng B., Zhong X., Ostrikov K.K. (2021). Photoluminescence mechanism of carbon dots: Triggering high-color-purity red fluorescence emission through edge amino protonation. Nat. Commun..

[40] Zhao Y.Y., Qu S.N., Feng X.Y., Xu J.C., Yang Y., Su S.C., Wang S.P., Ng K.W. (2019). Tailoring the Photoluminescence Excitation Dependence of the Carbon Dots via an Alkali Treatment. J. Phys. Chem. Lett..

[41] Liu C., Zhang F., Hu J., Gao W., Zhang M. (2020). A Mini Review on pH-Sensitive Photoluminescence in Carbon Nanodots. Front. Chem..

[42] Yu P., Wen X., Toh Y.-R., Tang J. (2012). Temperature-Dependent Fluorescence in Carbon Dots. J. Phys. Chem. C.

[43] Jiao Y., Gao Y., Meng Y., Lu W., Liu Y., Han H., Shuang S., Li L., Dong C. (2019). One-Step Synthesis of Label-Free Ratiometric Fluorescence Carbon Dots for the Detection of Silver Ions and Glutathione and Cellular Imaging Applications. ACS Appl. Mater. Interfaces.

[44] Wu X., Wu L., Cao X., Li Y., Liu A., Liu S. (2018). Nitrogen-doped carbon quantum dots for fluorescence detection of Cu^2+^ and electrochemical monitoring of bisphenol A. RSC Adv..

[45] Bayati M., Dai J., Zambrana A., Rees C., Fidalgo de Cortalezzi M. (2018). Effect of water chemistry on the aggregation and photoluminescence behavior of carbon dots. J. Env. Sci..

[46] Pylypova O., Paliienko K., Topchylo A., Zaderko A., Lysenko V., Skryshevsky V. (2026). Dynamic light scattering for probing metal ion induced aggregation of carbon dots. Mater. Adv..

[47] Rodriguez-Loya J., Lerma M., Gardea-Torresdey J.L. (2023). Dynamic Light Scattering and Its Application to Control Nanoparticle Aggregation in Colloidal Systems: A Review. Micromachines.

