# Supplementary material for: Molecular Precursor Engineering of Lignin-Derived Carbon Dots for Multicolor Fluorescence and Metal-Ion Sensing
Source: Nanomaterials (Basel). 2026 Jul 28;16(15):931. doi: 10.3390/nano16150931 (PMC13467686; doi:10.3390/nano16150931)
Supplement: Supplementary file 1 [file nanomaterials-16-00931-s001.zip › nanomaterials-4446138-supplementary.pdf]

Supplementary Material

Molecular Precursor Engineering of Lignin-Derived Carbon Dots for Multicolor Fluorescence and Metal-Ion Sensing

Bole Ma [1,†], Huiqing Wei [1,†], Jiaqi Tan [1], Liheng Chen [1,2,3,*], Minting Liang [1] and Xueqing Qiu [1,2,3,*]

[1] Guangdong Provincial Key Laboratory of Plant Resources Biorefinery, School of Chemical Engineering and Light Industry, Guangdong University of Technology, Guangzhou 510006, China
[2] Guangdong Provincial Laboratory of Chemistry and Fine Chemical Engineering Jieyang Center, Jieyang 515200, China
[3] Guangdong Basic Research Center of Excellence for Ecological Security and Green Development in Guangdong-Hong Kong-Marco Greater Bay Area (GBA), Guangdong University of Technology, Guangzhou 510006, China
* Correspondence: lihengchen@gdut.edu.cn (L.C.); cexqqiu@scut.edu.cn (X.Q.)
† These authors contributed equally to this work.

1 Photoluminescence quantum yield and environmental stability

The PLQYs were determined using the comparative method described in Section 2.3 of the main text. For the pH-stability tests, the pH values of the CD dispersions were adjusted from 1 to 14 using HCl or NaOH aqueous solutions, and the fluorescence intensities were recorded under identical excitation conditions. Each pH condition was measured in triplicate, and the results shown in Figure 4a–c are presented as the mean ± SD (n = 3). For the thermal and light-stability tests, the CD dispersions were exposed to visible light at 4, 20, and 40 °C, and their fluorescence intensities were monitored over 48 h.

2 Metal-ion sensing experiments

The fluorescence responses of C-CDs, H-CDs, and P-CDs toward different metal ions were investigated in aqueous solution. In the selectivity tests, various metal ions, including Ca^{2+}, Fe^{3+}, Pb^{2+}, Al^{3+}, Co^{2+}, Ag^{+}, Ni^{2+}, Cu^{2+}, Mg^{2+}, K^{+}, and Fe^{2+}, were added to the CD dispersions at a final concentration of 10 mM. The fluorescence spectra were recorded under identical testing conditions. Based on the selectivity results, Fe^{3+}, Cu^{2+}, and Ag^{+} were selected as the target ions for C-CDs, H-CDs, and P-CDs, respectively. For quantitative detection, different concentrations of the corresponding target ions were added to the CD dispersions. The low-concentration ranges used for linear calibration were 0.4-10 μM for Fe^{3+} detection by C-CDs, 0.2-10 μM for Cu^{2+} detection by H-CDs, and 0.4-10 μM for Ag^{+} detection by P-CDs. The limit of detection (LOD) was calculated using the equation:

$LOD = 3\sigma/k$

where σ is the standard deviation obtained from 10 repeated blank measurements and k is the slope of the corresponding low-concentration calibration curve. For the low-concentration Stern–Volmer analysis, the fluorescence data were evaluated using $F_0/F = 1 + KSV,app[Q]$, where F_0 and F are the fluorescence intensities in the absence and presence of the quencher, respectively, [Q] is the quencher concentration, and KSV,app is the apparent Stern–Volmer constant. The fitting was restricted to the analytical concentration ranges because the data at higher concentrations showed non-ideal Stern–Volmer behavior.

3 Detection in real water samples

The practical sensing performance of the CDs was evaluated in deionized water, tap water, and simulated industrial wastewater. The corresponding target ions, Fe^{3+}, Cu^{2+}, and Ag^{+}, were added to C-CDs, H-CDs, and P-CDs dispersions, respectively. The tested ion concentrations included 1 μM, 4 μM, 10 μM, 0.5 mM, 1 mM, 4 mM, and 10 mM. The fluorescence spectra were recorded under the same testing conditions and compared among different water matrices to evaluate the applicability of the CDs for metal-ion detection in practical aqueous environments. For the water-matrix experiments shown in Figure 5g–i, each condition was measured in triplicate, and the results are presented as the mean ± SD (n = 3). For the mixed-ion interference experiments, four conditions were compared for each probe: CDs alone, CDs with the mixed ions, CDs with the corresponding target ion, and CDs with both the mixed ions and target ion. The mixed-ion solution contained 1.0 mM each of $NaNO_3$, KNO_3, $MgSO_4$, and $Ca(NO_3)_2$.

4 Mixed-ion interference tests

A mixed-ion background containing 1.0 mM $NaNO_3$, 1.0 mM KNO_3, 1.0 mM $MgSO_4$, and 1.0 mM $Ca(NO_3)_2$ was used to evaluate matrix interference. Four conditions were examined for each sensing system: CDs alone, CDs with the mixed-ion background, CDs with the corresponding target ion, and CDs with both the mixed-ion background and the target ion. Fe^{3+}, Cu^{2+}, and Ag^{+} were used as the target ions for C-CDs, H-CDs, and P-CDs, respectively. The fluorescence spectra were recorded under identical conditions. Quenching efficiency was calculated as $(F_0 - F)/F_0 \times 100\%$.

5 Time-resolved photoluminescence measurements

The fluorescence decay curves of C-CDs/C-CDs–Fe^{3+}, H-CDs/H-CDs–Cu^{2+}, and P-CDs/P-CDs–Ag^{+} were recorded under matched excitation conditions. The decay curves were fitted using a biexponential model, $I(t) = B_1\exp(-t/\tau_1) + B_2\exp(-t/\tau_2)$. The intensity-weighted average lifetime was calculated using $\tau avg = (B_1\tau_1^2 + B_2\tau_2^2)/(B_1\tau_1 + B_2\tau_2)$, where B_i and τ_i represent the pre-exponential factor and lifetime of the ith component, respectively.

6 XPS analysis of metal-treated CDs

Freeze-dried C-CDs, H-CDs, and P-CDs were separately redispersed in ultrapure water at a concentration of 0.15 mg mL^{-1}. Aqueous solutions of $FeCl_3$, $CuSO_4$, and $AgNO_3$ were added to the C-CDs, H-CDs, and P-CDs dispersions, respectively. The mixtures were incubated for 2 h at 25 °C under static and dark conditions.

After incubation, unbound metal ions were removed by dialysis using a membrane with a molecular-weight cutoff of 1000 Da for 48 h. The retained CD fractions were washed with ultrapure water 3 times and subsequently freeze-dried. The resulting samples were denoted as C-CDs/Fe^{3+}, H-CDs/Cu^{2+}, and P-CDs/Ag^{+}.

The relatively high metal-ion concentrations used for XPS sample preparation were selected to obtain detectable metal-associated signals after removal of the unbound ions and were not used for evaluating the analytical sensing performance of the probes.

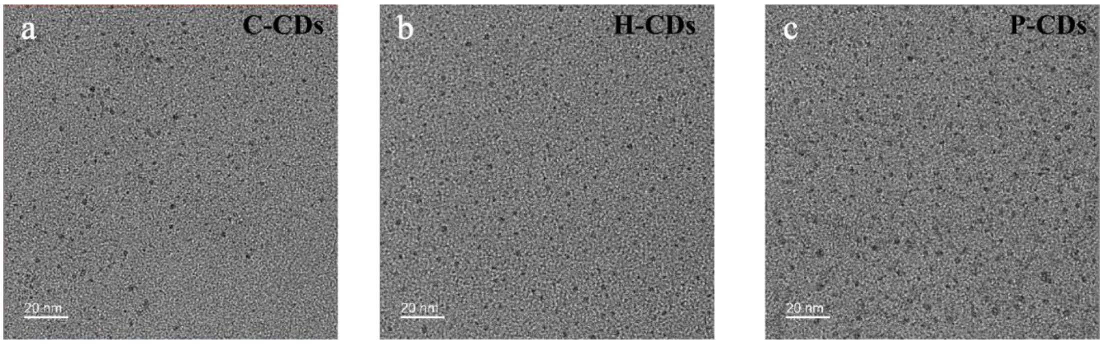

Figure S1. TEM images of (a) C-CDs, (b) H-CDs, and (c) P-CDs.

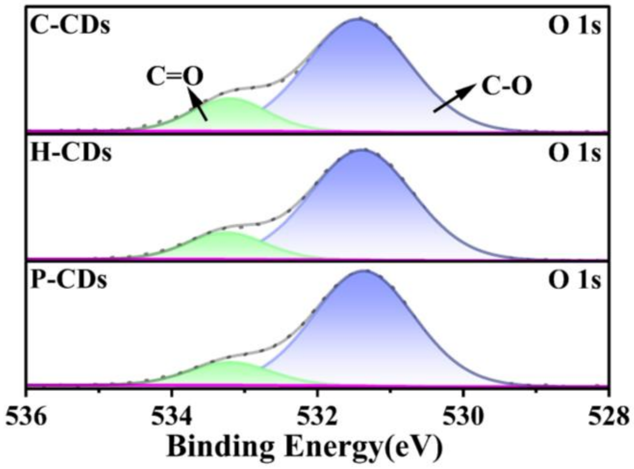

Figure S2. XPS high-resolution fitted O 1s spectra of C-CDs, H-CDs, and P-CDs.

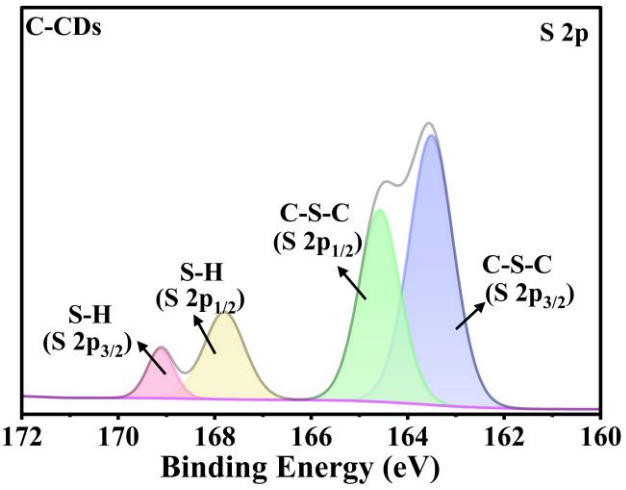

Figure S3. XPS high-resolution fitted S 2p spectrum of C-CDs.

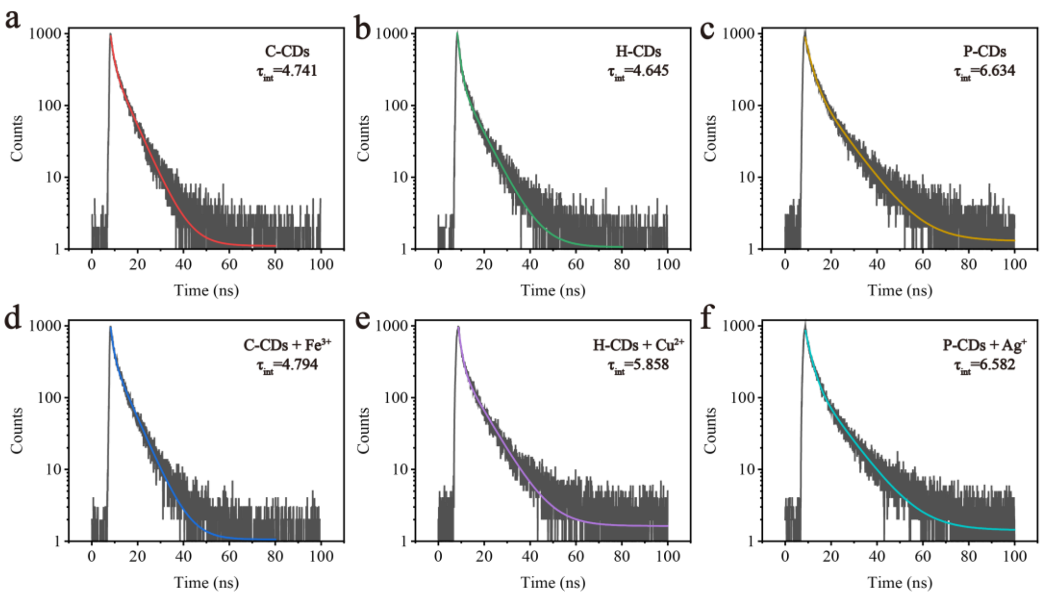

Figure S4. Time-resolved photoluminescence decay curves and corresponding biexponential fits of (a) C-CDs, (b) H-CDs, (c) P-CDs, (d) C-CDs after Fe^{3+} addition, (e) H-CDs after Cu^{2+} addition, and (f) P-CDs after Ag^{+} addition.

The average lifetimes of C-CDs/Fe^{3+} and P-CDs/Ag^{+} remained nearly unchanged after target-ion addition, whereas H-CDs/Cu^{2+} exhibited a redistribution toward the long-lived component.

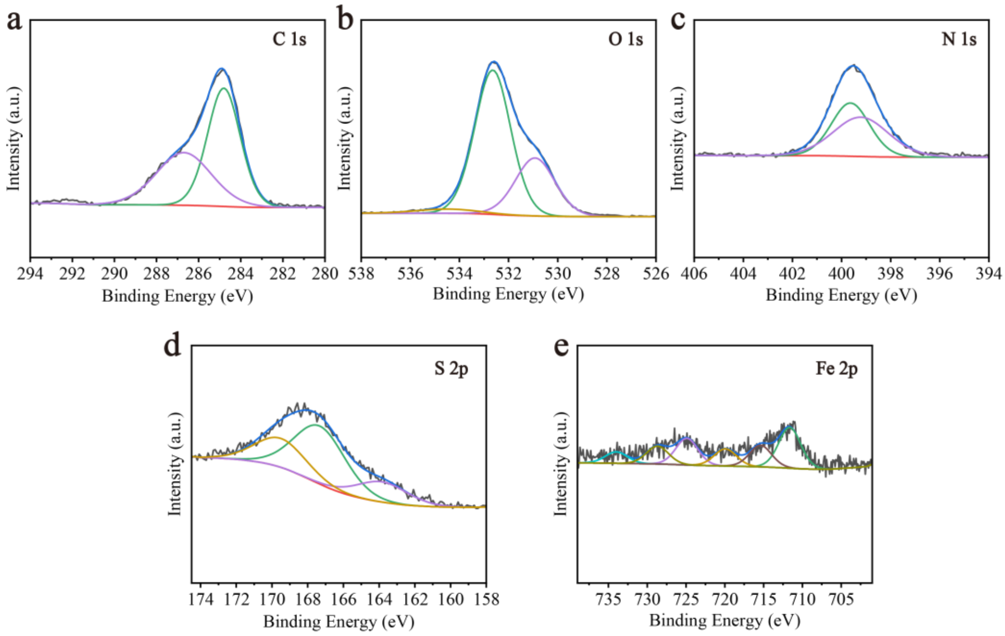

Figure S5. Charge-corrected high-resolution XPS spectra and peak deconvolution of Fe^{3+}-treated C-CDs: (a) C 1s, (b) O 1s, (c) N 1s, (d) S 2p, and (e) Fe 2p.

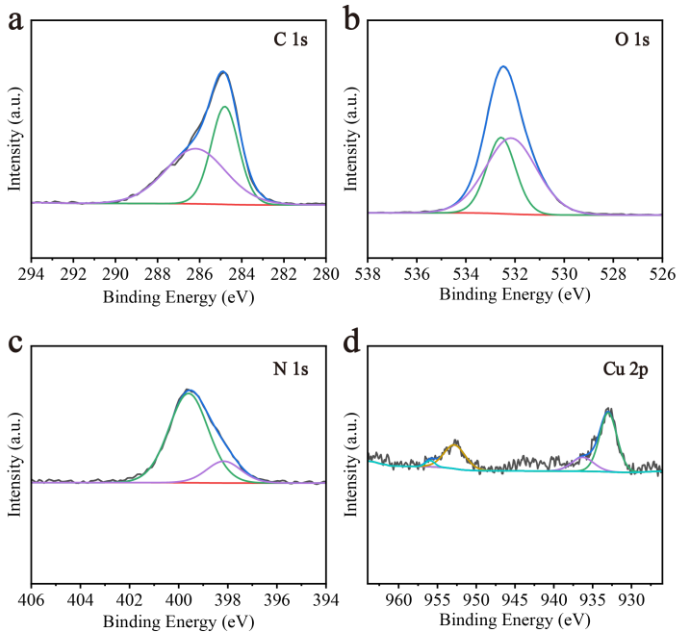

Figure S6. Charge-corrected high-resolution XPS spectra and peak deconvolution of Cu^{2+}-treated H-CDs: (a) C 1s, (b) O 1s, (c) N 1s, and (d) Cu 2p.

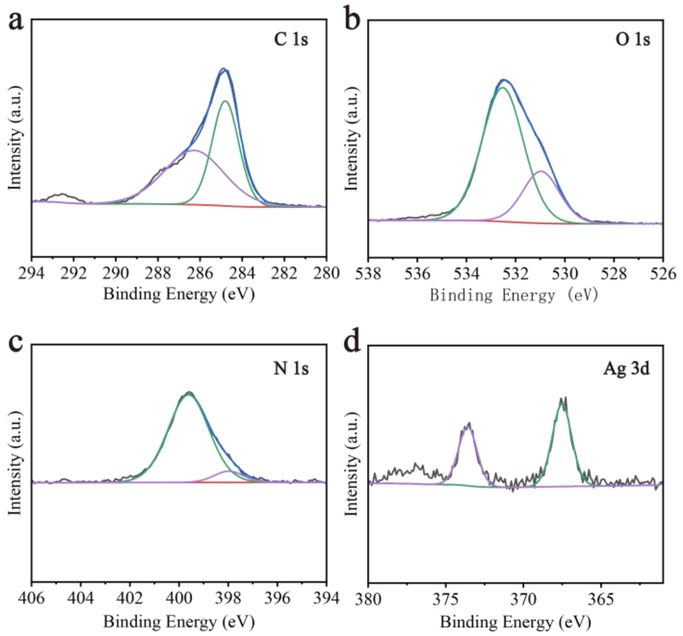

Figure S7. Charge-corrected high-resolution XPS spectra and peak deconvolution of Ag^{+}-treated P-CDs: (a) C 1s, (b) O 1s, (c) N 1s, and (d) Ag 3d.

Across Figures S5–S7, the changes in heteroatom-related components together with the newly detected metal signals support the association of the target ions with the CD surfaces. Because the C–C/C=C component was fixed at 284.8 eV for charge correction, it was not used independently to infer an unchanged carbon core.

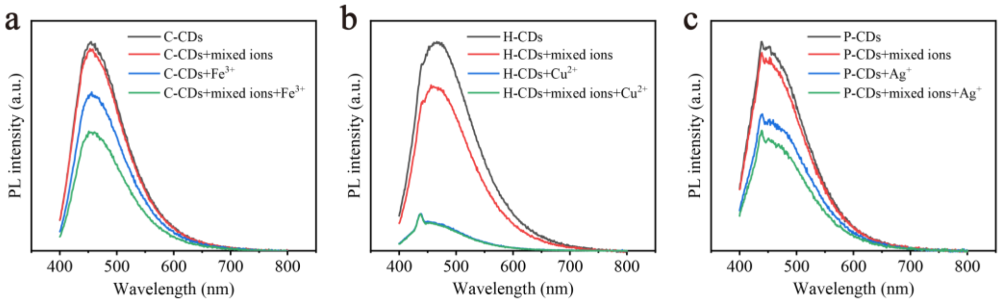

Figure S8. Normalized fluorescence emission spectra of (a) C-CDs/Fe^{3+}, (b) H-CDs/Cu^{2+}, and (c) P-CDs/Ag^{+} under single-ion and mixed-ion conditions. The spectra were recorded under 380 nm excitation, background-corrected, and normalized to the maximum fluorescence intensity of the corresponding blank CD dispersion.

The target-ion responses remained distinguishable in the mixed-ion background; however, the changes in the baseline fluorescence and net quenching efficiencies demonstrate probe-dependent matrix effects, as quantified in Table S6.

Table S1. Reaction conditions, fluorescence quantum yield, and product yield of different CDs.

Sample	Lignin (g)	K (g)	Oxalic acid (g)	C (g)	H (g)	P (g)	Reaction condition	PLQY	Yield
C-CDs	0.30	1.50	2.40	0.20	-	-	180 °C 6 h	29.62%	31.80%
H-CDs	0.60	1.50	2.40	-	0.60	-	180 °C 6 h	50.98%	33.13%
P-CDs	0.30	1.50	2.40	-	-	0.30	180 °C 6 h	40.78%	11.18%

Note: K, C, H, and P denote lysine, cysteine, histidine, and p-phenylenediamine, respectively.

Table S2. Summary of selected XPS data for C-CDs, H-CDs, and P-CDs.

Sample	C 1s Deconvolution (at.%)			N 1s Deconvolution (at.%)			O 1s Deconvolution (at.%)	
	C-C/C=C	C-O/C-N	C=O/O-C=O	Graphitic N	$-NH_2$	Pyridinic N	C=O	C-O
C-CDs	44.07	11.58	13.86	1.52	8.81	-	16.28	3.44
H-CDs	49.83	10.07	8.10	3.13	9.06	0.28	15.96	2.93
P-CDs	46.13	6.02	13.54	1.33	13.33	-	16.68	2.71

Table S3. Analytical performance parameters of C-CDs, H-CDs, and P-CDs for metal ion detection.

System	Linear range	R^2	KSV,app	LOD (μM)
C-CDs/Fe^{3+}	0.4-10μM	0.9085	8.15×10^2 M^{-1}	0.26
H-CDs/Cu^{2+}	0.2-10μM	0.9571	4.13×10^3 M^{-1}	0.05
P-CDs/Ag^{+}	0.4-10μM	0.9659	1.19×10^3 M^{-1}	0.11

The LOD was calculated using LOD = 3σ/k, where σ is the standard deviation obtained from 10 repeated blank measurements and k is the slope of the corresponding low-concentration calibration curve.

Table S4. Biexponential fitting parameters of the fluorescence decay curves of the pristine and target-metal-treated CDs.

Sample	τ_1 (ns)	Contribution 1 (%)	τ_2 (ns)	Contribution 2 (%)	τ_{avg} (ns)	χ^2
C-CDs	1.289 ± 0.040	25.87	5.946 ± 0.059	74.13	4.741 ± 0.061	0.942
C-CDs + Fe^{3+}	1.162 ± 0.038	23.16	5.888 ± 0.055	76.84	4.794 ± 0.058	0.979
H-CDs	1.229 ± 0.024	38.93	6.822 ± 0.080	61.07	4.645 ± 0.075	1.019
H-CDs + Cu^{2+}	1.336 ± 0.030	30.52	7.844 ± 0.076	69.48	5.858 ± 0.077	1.143
P-CDs	2.489 ± 0.048	43.18	9.784 ± 0.129	56.82	6.634 ± 0.113	1.084
P-CDs + Ag^{+}	2.324 ± 0.044	43.78	9.896 ± 0.134	56.22	6.582 ± 0.116	1.149

The decay curves were fitted using $I(t) = A + B_1\exp(-t/\tau_1) + B_2\exp(-t/\tau_2)$. The fractional contributions were calculated from $B_i\tau_i/\Sigma B_i\tau_i \times 100\%$. τ_{avg} is the intensity-weighted average lifetime. The uncertainties represent fitting standard errors rather than standard deviations from independent replicate measurements.

Table S5. Selected XPS binding energies of the pristine and target-metal-treated CDs.

Sensing system	Core level/component	Pristine CDs (eV)	Metal-treated CDs (eV)	ΔBE (eV)	Remark
C-CDs/Fe^{3+}	C 1s, C–O/C–N	286.15	286.68	+0.53	Shift of the surface-group-related component
C-CDs/Fe^{3+}	Fe $2p_{3/2}$	ND	711.68	—	Newly detected metal-associated signal
C-CDs/Fe^{3+}	Fe $2p_{1/2}$	ND	724.88	—	Newly detected metal-associated signal
H-CDs/Cu^{2+}	N 1s, principal component	399.90	399.58	-0.32	Change in the N-containing surface environment
H-CDs/Cu^{2+}	Cu $2p_{3/2}$	ND	932.98	—	Newly detected metal-associated signal

H-CDs/Cu^{2+}	Cu $2p_{1/2}$	ND	952.78	—	Newly detected metal-associated signal
P-CDs/Ag^{+}	N 1s, principal component	399.95	399.58	-0.37	Change in the N-containing surface environment
P-CDs/Ag^{+}	Ag $3d_{5/2}$	ND	367.48	—	Newly detected metal-associated signal
P-CDs/Ag^{+}	Ag $3d_{3/2}$	ND	373.58	—	Newly detected metal-associated signal

All binding energies were charge-corrected using the C–C/C=C component at 284.8 eV. ΔBE = BEmetal-treated − BEpristine. ND indicates that the corresponding metal signal was not detected in the pristine CDs. The Ag 3d spin-orbit splitting was approximately 6.10 eV. Because the pristine and metal-treated spectra were not always fitted using identical component numbers, only directly comparable representative surface peaks are listed; changes in the remaining envelopes are discussed qualitatively.

Table S6. Quantitative fluorescence responses of the three sensing systems under mixed-ion interference conditions.

Sensing system	F_{mixed}/F_0	F_{target}/F_0	$F_{mixed+target}/F_0$	Mixed-ion baseline effect (%)	Target quenching without mixed ions (%)	Net target quenching in the mixed-ion background (%)
C-CDs/Fe^{3+}	0.955	0.747	0.567	4.5	25.3	40.6
H-CDs/Cu^{2+}	0.779	0.127	0.126	22.1	87.3	83.8
P-CDs/Ag^{+}	0.947	0.648	0.575	5.3	35.2	39.3

F_0, F_{mixed}, F_{target}, and $F_{mixed+target}$ represent the fluorescence intensities of the CDs alone, CDs with mixed ions, CDs with the target ion, and CDs with both mixed ions and the target ion, respectively. The mixed-ion baseline effect was calculated as $(1 - F_{mixed}/F_0) \times 100\%$. Target quenching without mixed ions was calculated as $(1 - F_{target}/F_0) \times 100\%$. Net target quenching in the mixed-ion background was calculated as $(1 - F_{mixed+target}/F_{mixed}) \times 100\%$. Target-ion concentrations were identical in the corresponding single-ion and mixed-ion comparisons.
